# New Light on the Systematics of Fungi Associated with Attine Ant Gardens and the Description of *Escovopsis kreiselii* sp. nov.

**DOI:** 10.1371/journal.pone.0112067

**Published:** 2015-01-24

**Authors:** Lucas A. Meirelles, Quimi V. Montoya, Scott E. Solomon, Andre Rodrigues

**Affiliations:** 1 Department of Biochemistry and Microbiology, UNESP Univ Estadual Paulista, Rio Claro, SP, Brazil; 2 Department of Biosciences, Rice University, Houston, TX, United States of America; Emory University, UNITED STATES

## Abstract

Since the formal description of fungi in the genus *Escovopsis* in 1990, only a few studies have focused on the systematics of this group. For more than two decades, only two *Escovopsis* species were described; however, in 2013, three additional *Escovopsis* species were formally described along with the genus *Escovopsioides*, both found exclusively in attine ant gardens. During a survey for *Escovopsis* species in gardens of the lower attine ant *Mycetophylax morschi* in Brazil, we found four strains belonging to the pink-colored *Escovopsis* clade. Careful examination of these strains revealed significant morphological differences when compared to previously described species of *Escovopsis* and *Escovopsioides*. Based on the type of conidiogenesis (sympodial), as well as morphology of conidiogenous cells (percurrent), non-vesiculated conidiophores, and DNA sequences, we describe the four new strains as a new species, *Escovopsis kreiselii* sp. nov. Phylogenetic analyses using three nuclear markers (Large subunit RNA; translation elongation factor 1-alpha; and internal transcribed spacer) from the new strains as well as available sequences in public databases confirmed that all known fungi infecting attine ant gardens comprise a monophyletic group within the Hypocreaceae family, with very diverse morphological characteristics. Specifically, *Escovopsis kreiselii* is likely associated with gardens of lower-attine ants and its pathogenicity remains uncertain.

## Introduction

The diversity of microbial symbionts found within nests of attine ants, as well as the complex relationships among the various participants, makes the attine ant-microbial association among the most complex multipartite interactions known in nature. The tribe Attini comprises 257 ant species within 16 extant genera, including the recently described *Cyatta abscondita*, and one ichnogenus (*Attaichnus* Laza) [1–3]. As has been known for over a century, attine ants cultivate mutualistic basidiomycetous fungi as their primary food source [4]. During the 50 million-year evolutionary history of this tribe, five distinct types of agriculture have arisen: (i) lower attine agriculture (Agaricaceae); (ii) coral fungus agriculture (Pterulaceae); (iii) yeast agriculture (Agaricaceae); and (iv) higher attine ant agriculture, which includes (v) leaf-cutter ant agriculture (Agaricaceae) [1]. These groups differ in the type of substrates used for fungal cultivation, including insect frass, seeds, plant sap, arthropod exoskeletons, fresh leaves, and/or flower parts [5].

The last three decades have revealed that the fungal cultivars are not the only microbial symbionts that have evolved intimate relationships with attine ants. In 1999, Currie et al. [6] demonstrated the existence of a specific parasite that attacks the mutualistic cultivar. Fungi in the genus *Escovopsis* were described as the third member of the attine ant-fungal association. Subsequent studies have focused on evolutionary aspects of *Escovopsis* parasitism [7–11] and it was also demonstrated that the parasite attacks and consumes the cultivar mycelia [12]. Much of this work has focused on leaf-cutting ants, the most derived group within higher attine agriculture [7, 13–16].

Despite becoming a model system for the study of co-evolution and host-parasite dynamics, until recently little attention has been paid to the taxonomy of these fungi. In the 1990s, when the genus *Escovopsis* was proposed, only two species were known: *Escovopsis weberi* [17] and *E*. *aspergilloides* [18]. In 2013, three additional *Escovopsis* species—*E*. *microspora*, *E*. *moelleri* and *E*. *lentecrescen*s—were described, and a new genus, *Escovopsioides*, was proposed [19].

The five ex-type strains of *Escovopsis* species and *Escovopsioides nivea* (the only described species in the genus), were isolated from gardens of ants that engage in higher attine (including leafcutter) agriculture (i.e. groups (iv) and (v), respectively), which includes the ant genera *Sericomyrmex*, *Trachymyrmex*, *Acromyrmex* and *Atta* [1]. Numerous studies have confirmed that leaf-cutting ants (*Acromyrmex* and *Atta*) cultivate closely related, or even identical, strains of the mutualistic fungus [20–23], which are derived from the fungi cultivated by the higher attine genera *Trachymyrmex* and *Sericomyrmex* [24–26]. Nevertheless, no taxonomic study has been carried out on the parasites that infect gardens of attine ants that perform lower attine agriculture (i.e. group (i)); these fungi were separated into groups depending on their macroscopic characteristics, especially their colony (spore) color (brown, yellow, white and pink) [8–9], but no distinct species has been described in these groups.

We sampled fungus gardens of the lower attine ant *Mycetophylax morschi* and found four strains of a fungus species that are morphologically and phylogenetically distinct from previously described species of *Escovopsis* and *Escovopsioides*. The strains are described as a new species, *Escovopsis kreiselii*, that exhibits percurrent conidiogenous cells with sympodial conidiogenesis; such morphological characteristics may be considered less derived when compared to morphological aspects exhibited by other *Escovopsis* species described to date (including the presence of phialides in vesiculated conidiophores). Phylogenetic analyses of three nuclear loci confirmed the close relationship between *E*. *kreiselii* and the previously described species in a clade of Hypocreaceae fungi associated with attine ants.

## Materials and Methods

### Ethics statement

Collections made in the first expedition were conducted under collecting permit number 14789–1 issued by the “Instituto Brasileiro do Meio Ambiente e dos Recursos Naturais Renováveis” (IBAMA) to SES. Collections made in the second expedition were carried out under collecting permit number 31534 issued by the “Instituto Chico Mendes de Conservação da Biodiversidade” (ICMBio) to AR. LAM and QVM joined the second expedition and their names were included in permit 31534. No endangered or protected species were involved in this study.

### Nomenclature

The electronic version of this article in Portable Document Format (PDF) in a work with an ISSN or ISBN will represent a published work according to the International Code of Nomenclature for algae, fungi, and plants, and hence the new names contained in the electronic publication of a PLOS ONE article are effectively published under that Code from the electronic edition alone, so there is no longer any need to provide printed copies.

In addition, new names contained in this work have been submitted to MycoBank from where they will be made available to the Global Names Index. The unique MycoBank number can be resolved and the associated information viewed through any standard web browser by appending the MycoBank number contained in this publication to the prefix http://www.mycobank.org/MB/. The online version of this work is archived and available from the following digital repositories: [PubMed Central, LOCKSS].

### Sampling site and fungal isolation

Sampling was conducted during two different expeditions to Florianópolis, Santa Catarina, Brazil. In March of 2009, we isolated *E*. *kreiselii* (strain LESF53) from a nest of *M*. *morschi* found at Praia da Joaquina (nest AR090306–01, GPS: 27°37′50.01″S 48°27′3.64″W, elev. 1 m). In February 2014, we isolated three additional strains (LESF303, LESF304 and LESF305) of *E*. *kreiselii*, all associated with *M*. *morschi*. Strain LESF305 was isolated from a nest located at Praia da Joaquina (nest AR140226–01, GPS: 27°37′49.62″S 48°27′3.6″W, elev. 1.7 m) and the strains LESF303 and LESF304 were isolated from a single nest in Praia de Moçambique, on the northeast shore of the island of Florianópolis (nest AR140227–05, GPS: 27°31′24.96″S 48°25′3.78″W, elev. 1m). All nests were located in a coastal sand dune environment, a typical habitat of *M*. *morschi* [2, 27]. The whole fungus garden was sampled along with tending workers using a sterile spatula. Fungus gardens were kept in UV-sterilized plastic containers until they reached the laboratory at UNESP in Rio Claro, São Paulo, Brazil. Worker ants were subsequently identified by Dr. Rodrigo Feitosa and deposited in the entomological collection “Padre Jesus Santiago Moure” at Universidade Federal do Paraná, Curitiba, Brazil.

Small (0.5–1 mm in diameter) fragments of the fungus garden were plated on potato dextrose agar (PDA) supplemented with chloramphenicol [150 mg L^-1^, Sigma] and incubated at 25°C in the dark for seven days. Plates were monitored daily for fungal growth. Once filamentous fungi emerged from garden fragments, mycelia were subcultured on new PDA plates. Monosporic cultures were obtained for each strain and cultures were stored in 10% glycerol at -80°C at UNESP–Microbial Resource Center.

### Morphological characterization

Four different culture media were used for measuring the radial growth and to determine colony morphology: potato-dextrose agar (PDA), malt extract agar 2% (MA2%), cornmeal agar (CMD) and synthetic nutrient agar (SNA). Strains were cultivated in triplicates at four different temperatures for each medium (10, 20, 25, 30 and 35°C). Microscopic morphological characters were examined on slide culture preparations. For this, we placed a 5 mm^2^ block of PDA on a microscopic slide and inoculated the fungus; after that, the material was covered with a cover slip and incubated at 25°C for seven days. Then, slides were examined under a light microscope. Conidia, conidiophores and conidiogenous cells were measured using the software LAS EZ (Leica Application Suite) using measurements of 30 replicates for each structure.

To visualize these same structures using scanning electron microscopy, fungus samples were fixed in osmium tetroxide vapor for 72 h, dehydrated sequentially through a series of acetone concentrations (50, 75, 90, 95 and 100%) and then dried until the critical point using liquid CO_2_ (Balzers CPD030). The dried material was mounted in stubs, sputtered with gold (Balzers SCD050) and examined with a scanning electron microscope (TM3000, Hitachi).

### DNA extraction, PCR amplification and sequencing

Genomic DNA was extracted from mycelia grown on PDA for 7 days following a modified version of the CTAB method [8, 28]. Three different nuclear DNA markers were amplified for phylogenetic analysis: (i) large subunit RNA—LSU; (ii) translation elongation factor 1-alpha—*tef1*; and (iii) internal transcribed spacer—ITS. The primers and conditions used for amplification are described in Table 1. PCR products were cleaned up with *Kit Wizard SV Gel and PCR Clean-up System* (Promega) following the manufacturer’s protocol. Cycle sequencing reactions were carried out with 20 ng of template using *BigDye Terminator v*. *3*.*1 Cycle Sequencing Kit* (Life Technologies) following the manufacturer instructions. Sequences were generated using ABI3500 (Life Technologies). Contigs were assembled in Bioedit v. 7.1.3 [29] and queried using the NCBI—GenBank to find the closest known relatives. Sequences of strain LESF53 were deposited in GenBank under accessions KJ808765—KJ808767. We also sequenced the partial *tef1* gene for *Escovopsis microspora* CBS 135751^T^, which was deposited in GenBank under accession KJ935030.

**Table 1:** Molecular markers, primers and PCR conditions used in this study.

Marker	Primers	Conditions
LSU	CLA-F (5’- GCATATCAATAAGCGGAGGA); CLA-R (5’- GACTCCTTGGTCCGTGTTTCA) [7]	2 min of denaturation at 95°C, 40 cycles consisting of 30 s at 95°C, 60 s at 62°C, 90 s at 72°C and 5 min of extension at 72°C [19]
tef1	EF6–20F (5’-AAGAACATGATCACTGGTACCT-3’); EF6–1000R (5’-CGCATGTCRCGGACGGC-3’) [10]	96°C for 3 min, 35 cycles at 96°C for 30 s, 61°C for 45 s and a final extension step at 72°C for 1 min [53]
ITS	ITS4 (5’-TCCTCCGCTTATTGATATGC-3’); ITS5 (5’-GGAAGTAAAAGTCGTAACAAGG-3’) [54]	96°C for 3 min, 35 cycles at 94°C for 1 min, 55°C for 1 min and a final extension step at 72°C for 2 min [54]

### Phylogenetic analyses

To determine the phylogenetic position of the new strains, three different phylogenetic reconstructions were performed: (i) a phylogeny within the Hypocreaceae family using LSU partial sequences; (ii) a phylogeny within the *Escovopsis* clade using *tef1* partial sequences (previously examined strains of these fungi have been sequenced for the gene *tef1* and their sequences are available in GenBank); and finally (iii), a phylogeny including only the formally described species of *Escovopsis* and *Escovopsioides* using concatenated sequences of ITS and *tef1*.

Alignments were obtained independently for each molecular marker using MAFFT v.7 [30] and nucleotide substitution models were selected using the Akaike information criterion (AIC) with a confidence interval of 95% in jModelTest 2 [31]. Independent runs in jModelTest 2 were performed for each molecular marker of the concatenated analysis (analysis number iii). Phylogenies were reconstructed using maximum likelihood (ML) in RAxML v.8 [32] and Bayesian Inference (BI) in MrBayes v.3.2.2 [33].

For positioning our strain within the Hypocreaceae, we first reconstructed the phylogeny of several species and genera belonging to this family using the LSU partial sequences. We selected 23 sequences used by Augustin et al. [19] and rooted using two members of Clavicipitaceae. We reconstructed 1000 independent ML trees under the GTR+I+G model in RAxML, and kept the tree with the best score. We evaluated the reliability of the tree topology by performing 2000 bootstrap replicates (bootstrap values converged at 1150 replicates [34]). Bayesian inference also used the GTR+I+G model of nucleotide substitution in two independent runs in MrBayes, each with three heated chains and one cold chain; each run consisted of Markov Chain Monte Carlo (MCMC) sampling for 2 million generations. Convergence occurred when the standard deviation (SD) of split frequencies fell below 0.01; the first 25% of MCMC generations were discarded as burn-in.

To further refine the position of the new strains within the *Escovopsis* clade, we used phylogenetic inference for *tef1* partial sequences using the same conditions described above for both ML and BI analyses. In this case, bootstrap values converged after 850 replicates. Two million MCMC generations were again sufficient for achieving SD of split frequencies of less than 0.01. This tree was rooted using three sequences of other Hypocreaceae (*Hypomyces polyporinus*, *Hypocrea lutea* and *Trichoderma* sp.) used in previous studies [7, 9].

A third analysis used concatenated alignments of ITS and *tef1* sequences generated using Winclada v.1.00.08 [35]. A partitioned ML analysis was conducted in RAxML using GTR+G for each partition. The alpha shape parameter of the gamma model of rate heterogeneity, empirical base frequencies, and evolutionary rates in the GTR matrix were estimated independently for each partition [32]. As before, 1000 ML trees were reconstructed and 2000 bootstraps replicates were performed (bootstraps values converged at 1950 replicates). For BI, a partitioned analysis was performed in MrBayes under GTR+G for each partition. The number of independent runs, number of chains, and the burn-in were the same as described above, but each run consisted of 1 million MCMC generations, which was sufficient to reach a SD of split frequencies of less than 0.01. We selected three other Hypocreaceae species (*Trichoderma hamatum*, *T*. *pubens* and *Hypocrea rufa*) that had ITS and *tef1* sequences available in GenBank (NCBI) as the outgroup. The final trees were edited in FigTree v1.4.0 (http://tree.bio.ed.ac.uk/software/figtree/) and Adobe Illustrator CS6 (Adobe Systems).

## Results

### Morphology

**Figure 1:**
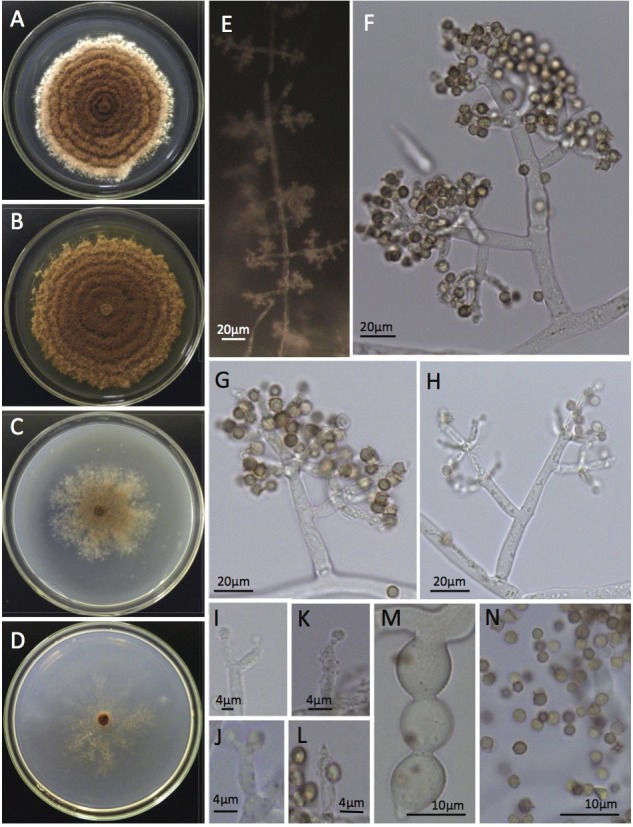
General morphological characteristics of *Escovopsis kreiselii* CBS 139320 (= LESF53). A-D: cultures grown in PDA, MA2%, CMD and SNA after 14 days at 25°C, respectively; E: Conidiophores pattern formation on the aerial mycelia; F-H: Conidiophore branching pattern; I-L: Percurrent conidiogenous cells; M: Chlamydospores developed in chains and N: Globose to subglobose conidia.

**Figure 2:**
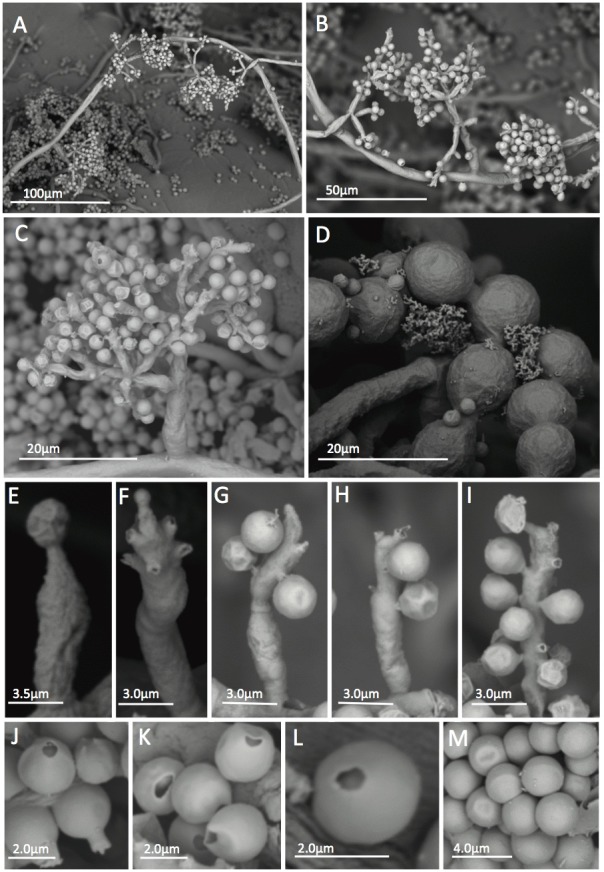
SEM of *Escovopsis kreiselii* CBS 139320 (= LESF53) showing morphological aspects. Slide cultures grown in PDA and SNA for five days at 25°C. A-B: Conidiophore growing patterns on the aerial mycelia; C: Conidiophore branching pattern; D: Chlamydospores; E-I: Percurrent conidiogenous cells showing conidia attached to the cells by denticles; J-M: Conidia. J: Conidia with attached denticle; K-L: Conidia without denticles and M: smooth walled conidia.

Morphological differences were observed between the *E*. *kreiselii* strains (LESF53, LESF303, LESF304 and LESF305) and all previously described species of *Escovopsis* and *Escovopsioides* associated with attine ants [19]. The most distinguishing feature of the new species compared with others is the type of conidiogenesis: *E*. *kreiselii* has holoblastic sympodial conidiogenesis with percurrent conidiogenous cells (Figs. 1 and 2). The colony color also differs from previously described species: *E*. *kreiselii* spores are pink while other described species of *Escovopsis* are brown, and *Escovopsioides* is white (Fig. 1). Such color differences appear to be important to distinguish fungi within the *Escovopsis* clade [6, 9, 19]. *Escovopsis kreiselii* grows better at 25°C (but also grows well at 20°C, see S1 Fig.) which is consistent with thermal preferences described for some attine ants [36]. Moreover, no growth was observed at 30°C, indicating that *E*. *kreiselii* is sensitive to high temperatures. *E*. *kreiselii* also has chlamydospores (Fig. 1M and 2D) similar to *Escovopsioides nivea*.

### Phylogenetic analyses

**Figure 3:**
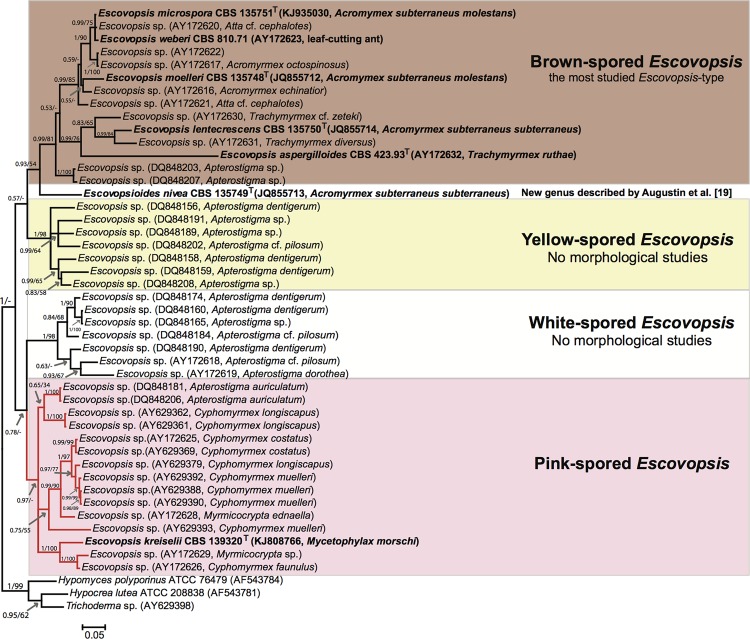
Phylogenetic position of *Escovopsis kreiselii* within the *Escovopsis* clade based on *tef1* sequences reconstructed using Bayesian Inference. All *Escovopsis* species described so far are denoted in bold. In addition to the newly described species, forty-three *tef1* sequences representing all *Escovopsis* morphotypes used in previous studies were retrieved from GenBank. Sequences of *tef1* from other Hypocreaceae were used as outgroup. The voucher accession numbers in culture collections follow the taxon names. GenBank accessions and the ant species from which the fungi were isolated are given in parentheses. Different colors indicate the fungal morphotypes found in the *Escovopsis* clade. Bootstrap values from ML analyses are also indicated from a similar topology. Only PP and bootstrap values ≥ 0.5 or 50 are shown. Phylogeny based on Gerardo et al. [9]. T: ex-type strains. Bar: 0.05 substitutions per nucleotide position.

**Figure 4:**
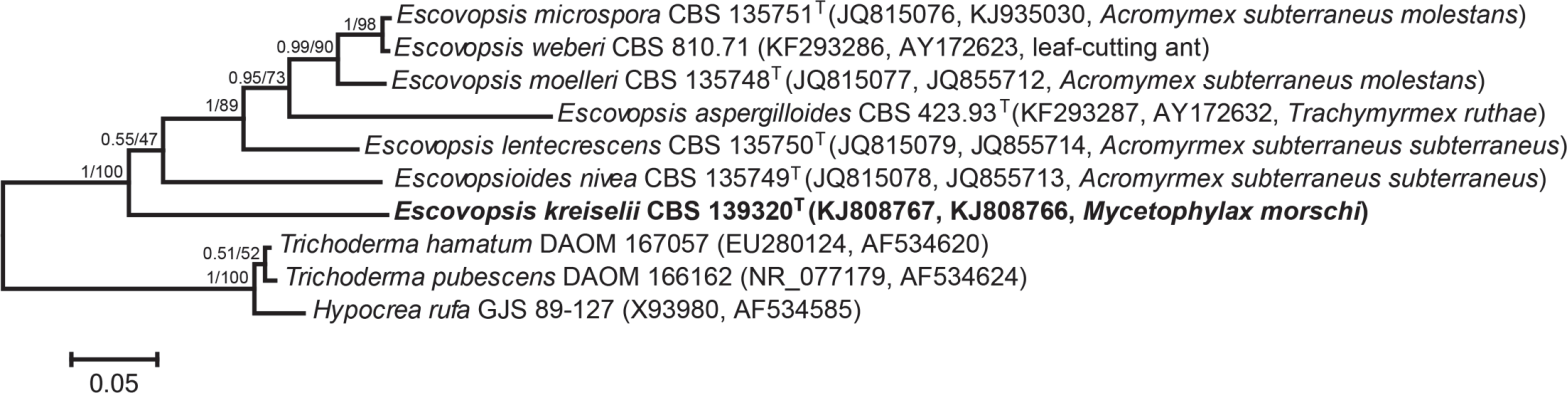
Phylogeny of described species within the *Escovopsis* clade. This tree was generated from Bayesian analyses of two different markers: ITS and *tef1*. Sequences of other Hypocreaceae species were used as outgroup. The voucher accession numbers in culture collections follow the taxon names, and GenBank accessions and the ant species from which the fungi were isolated are given in parentheses. The new species, *Escovopsis kreiselii* is denoted in bold. ML analyses resulted in an identical topology and ML bootstraps values are shown above nodes. T: ex-type strains. Bar: 0.05 substitutions per nucleotide position.

The tree topologies reconstructed using both ML and BI in three different phylogenetic analyses were very similar; the final Bayesian trees are displayed in Figs. 3, 4, and S2 Fig. with posterior probabilities and ML bootstrap values to indicate support for individual nodes. The LSU analysis grouped *E*. *kreiselii* as closely related to *Escovopsis* and *Escovopsioides* species within the phylogeny including other Hypocreaceae members. These fungi are grouped within an “attine-ants associated clade” (S2 Fig.). To compare the new strain with all *Escovopsis* diversity previously explored in other studies, we also performed a phylogenetic analysis using *tef1* (Fig. 3). In this phylogeny, *E*. *kreiselii* grouped within the clade of pink-colored *Escovopsis* described in Gerardo et al. [9]. Unfortunately, it is not possible to include more genes within this phylogenetic analysis because most studies on the parasite phylogeny used only *tef1* sequences and the strains are not available in public culture collections. Therefore, aiming to increase the reliability of our analysis, we also reconstructed a tree using two markers (ITS and *tef1*) of all described species of *Escovopsis* and *Escovopsioides*, similar to the analysis conducted by Augustin et al. [19]. Overall, our results suggest that *E*. *kreiselii* is a sister group to the clade that contains all previously described species of *Escovopsis* (Fig. 4), but the phylogenetic position of *Escovopsioides nivea* remains uncertain, varying depending on the molecular marker used (see Figs. 3, 4 and S2 Fig.). These results along with the morphological data support *E*. *kreiselii* as a new species within the hypocrealean fungi associated with attine ants.

### Taxonomy

**Emendation of the genus *Escovopsis* Muchovej & Della Lucia.** Augustin et al. [19] added information regarding the conidia morphology to the genus originally described by Muchovej and Della Lucia as *Escovopsis* [17]. However, until now, the genus is described as “*vesiculate; vesicles cylindrical to globose*, *evanescent; phialides hyaline*, *swollen at base*, *extending to a narrow neck; conidia in short basipetal chains*, *aseptate*, *hyaline at first becoming pigmented with an ornamented or mucilaginous brown outer coat or sheath*, *phoretic*” [19]. To accommodate morphological variations observed in *E*. *kreiselii*, we propose an emendation on the description of the genus *Escovopsis* as follows: non-vesiculated conidiophores are present in less derived strains, with non-phialidic conidigenous cells producing solitary conidia (as indicated in Figs. 1 and 2).

### *Escovopsis kreiselii* L.A. Meirelles, Q.V. Montoya, S.E. Solomon & A. Rodrigues sp. nov.

Mycobank: MB809176

*Colonies* on PDA, MA2%, CMD and SNA growing slowly, up to 5.3–6.0 cm, 6.6–8.1 cm, 3.9–4.5 cm and 4.3–5.2 cm in diameter after 14 days at 25°C. Mycelium reaching the edge of a 9 cm Petri dish in 24 and 22 days on PDA and MA2%, respectively, and after 30 days on CMD and SNA. Inconspicuous growth observed at 10°C; no growth at 30°C. Concentric growth observed in colonies cultured on PDA and MA2% (S1 Fig.). Hyaline hypha present in the aerial mycelia, stolon-like structures not observed. Colonies at first white, later attaining pink to brownish colors depending on the media (Fig. 1A-D). No soluble pigments observed in all media. Colony reverse is uncolored. Intercalary concatenated chlamydospores found in the submerged and in the aerial mycelia, 10.8–21.4 μm in length × 8.3–16.6 μm in width. *Conidiophores* arising from fertile aerial hypha in opposite and intercalary patterns (Fig. 1E and Fig. 2A-B), at first white and quickly becoming light to dark pink. A septum is often observed at the base of each conidiophore. Irregular conidiophore branching pattern, some have verticils (of 3 to 4 branches), some have 2 opposite branches and some are solitary; smooth walled, hyaline, 29.0–229.00 μm in length and 2.5–8.6 μm in diameter. *Conidiogenous cells* are holoblastic, formed at the apex of each branch, intercalary or solitary on the fertile hypha. Each branch of the conidiophore has up to 4–6 conidiogenous cells measuring 5.3–13.4 μm in length and 2.5–7.7 cm in width. Conidiogenous cells are percurrent, hyaline, arising in verticils often with numerous denticle-like structures (Figs 1K, 2E-I). Conidia are globose to subglobose, brown, 2.4–3.2 μm in diameter, thick walled with smooth or rough surface (Figs. 1N, 2C, and 2E-M). Conidia often bear denticle-like structures (Fig. 2J).

*Specimen examined*: BRAZIL. Santa Catarina, Florianópolis, Praia da Joaquina, GPS: 27°37′50.01″S; 48°27′3.64″W, elev. 1 m, Fungus garden, 03, 2009. *A*. *Rodrigues*. Ex-type strain LESF53 (= CBS 139320, = CBMAI 1691). Holotype: CBS H-22062 (dried culture on PDA).

*Additional specimens examined*: BRAZIL. Santa Catarina, Florianópolis, Praia de Moçambique, GPS: 27°31′24.96″S; 48°25′3.78″W, elev. 1 m, Fungus garden, 02, 2014. *A*. *Rodrigues*. LESF303 (= CBS 139321, = CBMAI 1692) and LESF304. BRAZIL. Santa Catarina, Florianópolis, Praia da Joaquina, GPS: 27°37′49.62″S; 48°27′3.6″W, elev. 1.7 m, Fungus garden, 02, 2014. *A*. *Rodrigues*. LESF305 (= CBS 139322, = CBMAI 1693).

*Sequences*: LSU: KJ808765, *tef1*: KJ808766, ITS: KJ808767

*Etymology*: Named in honor of Dr. Hanns Kreisel, who first described the fungal parasite *Phialocladus* currently known as *Escovopsis*. Kreisel did not designate a type strain for *Phialocladus* [37], thus this name was considered a *nomen invalidum* [17].

*Habitat*: isolated from fungus gardens of the attine ant *Mycetophylax morschi*.

## Discussion

The intricacy of symbioses between animals and microbes is emerging as a major theme in 21^st^ century biology (e.g. the human microbiome project). Despite a new appreciation of the ubiquity and importance of such symbioses, the attine ants and their fungal symbionts have long been recognized as an example of the complex interactions between animals and microbes. Although the fungal cultivar used as a food source for ants was first recognized in 1874 [38], it was not until the 1990s, and especially the 2000s, that the high fungal diversity associated with attine ants began to be recognized [6, 13, 17–19, 39–46].

The genus *Escovopsis* was likewise first observed in the 19^th^ century [47]. During the 20^th^ century, some early studies documented the presence of these fungi in attine ant nests [37, 48], but the genus and the first two species were not formally described until the 1990s [17, 18]. Recently, Augustin et al. [19] described three additional species within *Escovopsis* and the new genus *Escovopsioides* (originally studied by Möller in 1893 [47]; however he interpreted this genus to be a “weak form” of the fungus cultivated by leaf-cutting ants). Moreover, no taxonomic study was performed for *Escovopsis* associated with lower attine ants and the morphological aspects of these fungi are still poorly known.

Gerardo et al. [9] examined different *Escovopsis* morphotypes, documenting the existence of at least four different colored groups (pink, white, yellow and brown) associated with *Apterostigma* and *Cyphomyrmex* ants. However, that study aimed to explore the phylogenetic diversity of the fungus, and no detailed morphological examination was carried out. In our phylogenetic analyses, *E*. *kreiselii* falls within the pink-colored *Escovopsis* clade (Fig. 3), suggesting that species of the pink clade may exhibit significant morphological differences when compared to other described *Escovopsi*s species; nevertheless, detailed morphological analyses on these strains are necessary to confirm this hypothesis.

Currie et al. [6, 7] provided evidence that *Escovopsis* is a specific parasite of the mutualistic fungus cultivated by attine ants and that *Escovopsis* has co-evolved with the ants’ fungal cultivar, as evidenced by co-cladogenesis between species of both fungi. The morphological analyses conducted by Currie et al. [7] were not sufficient to identify detailed differences in conidiophore structure or conidiogenesis type for the distinct *Escovopsis* clades. Our study extends the findings of Currie et al. [7] and corroborates that *Escovopsis* is a genus that exhibits diverse morphological features. This concept is consistent with the known diversity of this group of ants [1, 3] and that of their mutualistic fungi [24, 26, 49]. Morphological characteristics of *E*. *kreiselii* support its distinction from other *Escovopsis* species due to the conidiogenesis type, which is sympodial in *E*. *kreiselii* and phialidic for the other *Escovopsis* described so far. Moreover, no vesicles were observed in the conidiophores of *E*. *kreiselii*, which is contrary to the vesiculated conidiophores found in other *Escovopsis* species. In addition, all the described *Escovopsis* species are brown, whereas *E*. *kreiselii* is pink. The conidiogenous cells of the new species are percurrent, contrary to the phialidic conidiogenous cells observed in *Escovopsis* from higher attines. Furthermore, *E*. *kreiselii* produces solitary conidia. Due to the inclusion of *E*. *kreiselii* in the genus *Escovopsis*, it is clear that this group exhibits very diverse morphological aspects and an extension of the genus is necessary to accommodate the new morphological diversity. Future taxonomic work will enhance our understanding of this diversity. Although previous phylogenetic approaches have hinted at the vast genetic diversity of *Escovopsis* [7, 9–11], detailed examination of the morphology of the yellow, white and other strains of the pink-spored *Escovopsis* is essential to better understand the systematics of this group. Such examination will also aid in studies regarding evolutionary and ecology of host-pathogen dynamics in this system.

An important event in the study of this group was the description of *Escovopsioides* by Augustin et al. [19]. However, the phylogenetic position of this taxon remains uncertain. In our LSU phylogeny, *Escovopsioides* was an outgroup to the clade consisting of all formally described *Escovopsis* species (S2 Fig.); nevertheless, in the phylogeny reconstructed with *tef1*, *Escovopsioides* is sister to the brown-spored *Escovopsis*, which together with the yellow-spored *Escovopsis* form a clade that is sister to the white-spored and pink-spored *Escovopsis* (Fig. 3). A similar topology was suggested by the concatenated (ITS and *tef1*) phylogeny, but with low statistical support for *Escovopsioides* position (Fig. 4). Under a morphological approach, *Escovopsioides* seems to be more similar to species described within the brown-spored *Escovopsis* group (both have phialides with conidia produced in chains). The main difference is that the phialides for the *Escovopsioides* are lageniform and produced on terminal and intercalary globose vesicles [19]. *Escovopsioides nivea* has no pigmentation while all *Escovopsis* species exhibiting phialidic conidiogenese are brown in color [19]. Moreover, the percurrent conidiogenous cells of *E*. *kreiselii* may be considered a less derived character when compared to the phialides of brown *Escovopsis* and *Escovopsioides*. Nevertheless, only one species of *Escovopsioides* is known, represented by a single DNA sequence. Thus, sampling more strains of *Escovopsioides* may resolve these incongruences in the phylogenetic reconstructions using different genes and clarify its position. If this fungus is in fact more related to the brown *Escovopsis* clade than to the other color clades (i.e. if it groups within the *Escovopsis* clade), future studies might reclassify *E*. *nivea* as an *Escovopsis* species, maintaining the monophyly of the hypocrealean fungi specialized in infecting gardens of attine ants. It seems that, over evolutionary time, this group of fungi developed different conidiogenesis types and such morphological aspects might indicate adaptations related to their interactions with the ants and/or the mutualistic cultivar.

Finally, as a member of the *Escovopsis* group, *E*. *kreiselii* is presumed to be a fungal parasite of attine fungal gardens; however, the pathogenicity of this species remains unknown. Currie [50] demonstrated that *Escovopsis* is a specific parasite of the mutualistic fungus that decreases garden biomass accumulation. Reynolds and Currie [12] suggested that *Escovopsis* attacks the mutualistic fungus using chemical compounds and Gerardo et al. [8] demonstrated specificity in host-parasite interactions among the pink-spored *Escovopsis*. Moreover, several studies have tested the *in vitro* pathogenicity of brown-spored *Escovopsis* of leaf-cutting ants [51, 52]. However, no study to date has considered this approach for the other *Escovopsis* clades. It is possible that *E*. *kreiselii* attacks the mutualistic fungus of *M*. *morschi*, but this hypothesis remains to be supported empirically. It is noteworthy that all the three *M*. *morschi* nests we sampled were apparently healthy, suggesting that *E*. *kreiselii* was either in a dormant state within the fungus gardens at the time of sampling or that *E*. *kreiselii* is not in fact a virulent pathogen of lower attine fungal gardens.

## Supporting Information

S1 Fig *Escovopsis kreiselii* CBS 139320 (= LESF53) mycelial growth after 14 days grown in different culture media at two temperatures. PDA: potato-dextrose agar; MA2%: malt extract agar 2%; CMD: cornmeal agar; SNA: synthetic nutrient agar.
(TIFF)

S2 Fig Phylogeny of Hypocreaceae reconstructed from the LSU gene indicating the position of *Escovopsis kreiselii*. The phylogeny of 22 Hypocreaceae species and two Clavicipitaceae species as outgroup was reconstructed using Bayesian Inference. The voucher accession numbers in culture collections follow the taxon names and the GenBank accession numbers are given in parentheses. The clade highlighted in gray represents fungi strictly associated with attine ants. Posterior probabilities of nodes are given along with ML bootstraps values for a similar topology; only PP and bootstrap values ≥ 0.5 or 50 are shown on branches, respectively. Phylogeny based on Augustin et al. [19]. Bar: 0.05 substitutions per nucleotide position. T: ex-type strains.
(TIFF)
