# Supplementary figures and images for: New Light on the Systematics of Fungi Associated with Attine Ant Gardens and the Description of Escovopsis kreiselii sp. nov
Source: PLoS One. 2015 Jan 24;10(1):e0112067. doi: 10.1371/journal.pone.0112067 (PMC4305282; doi:10.1371/journal.pone.0112067)

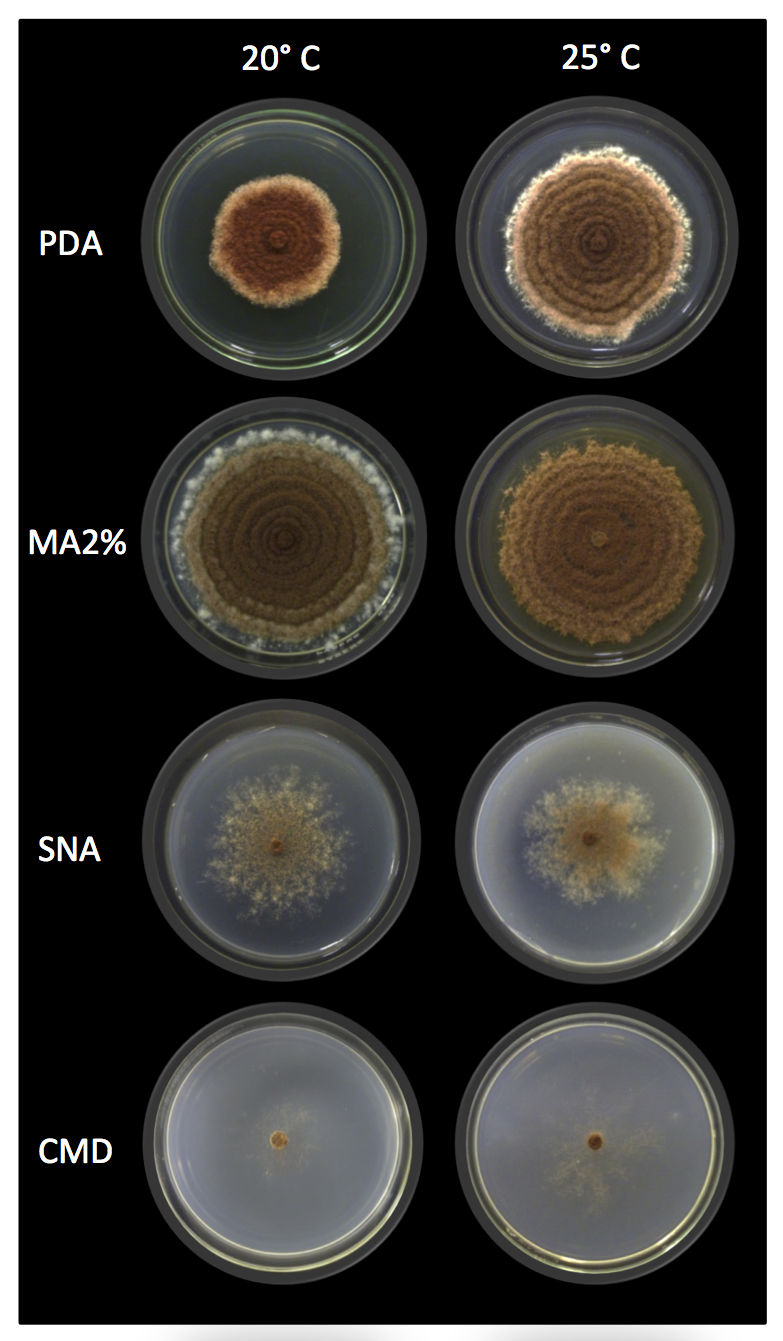

Supplement: S1 Fig — PDA: potato-dextrose agar; MA2%: malt extract agar 2%; CMD: cornmeal agar; SNA: synthetic nutrient agar. (TIFF) [file pone.0112067.s001.tiff]

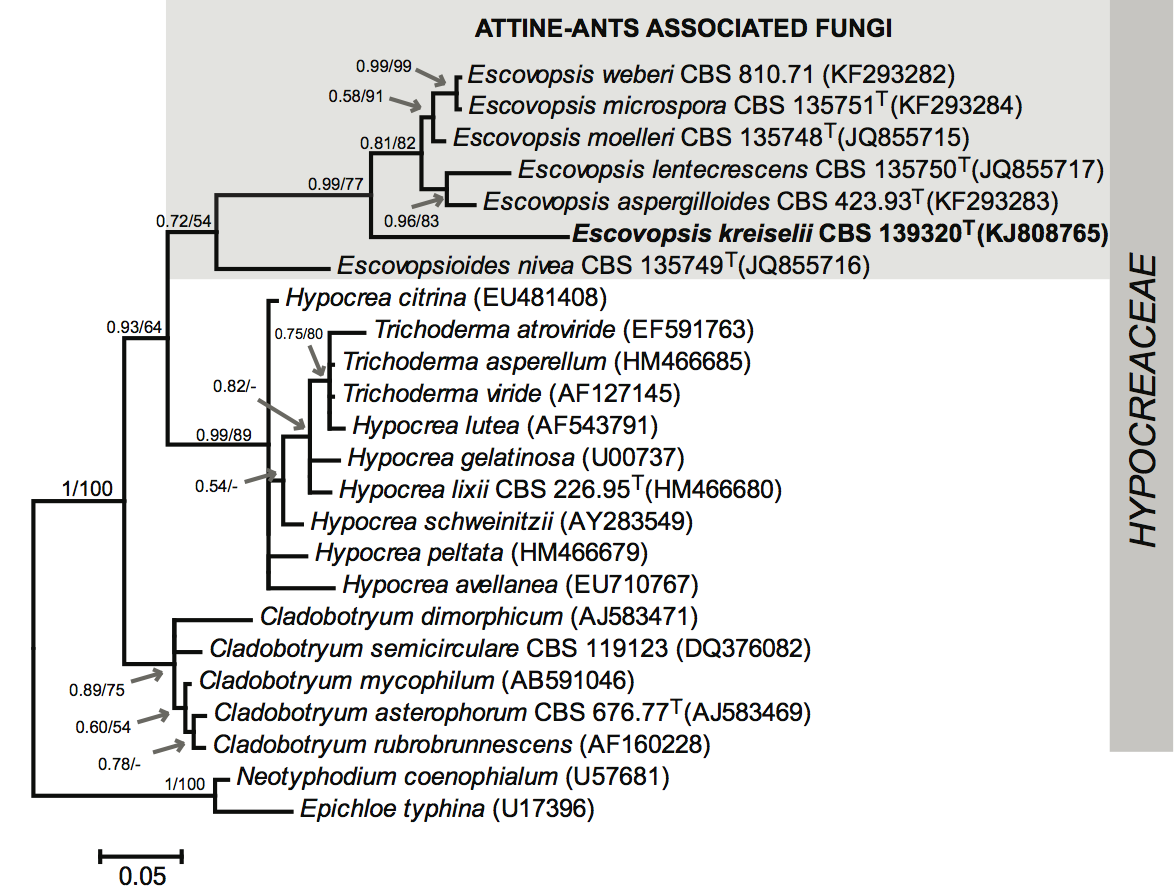

Supplement: S2 Fig — The phylogeny of 22 Hypocreaceae species and two Clavicipitaceae species as outgroup was reconstructed using Bayesian Inference. The voucher accession numbers in culture collections follow the taxon names and the GenBank accession numbers are given in parentheses. The clade highlighted in gray represents fungi strictly associated with attine ants. Posterior probabilities of nodes are given along with ML bootstraps values for a similar topology; only PP and bootstrap values ≥ 0.5 or 50 are shown on branches, respectively. Phylogeny based on Augustin et al. [19]. Bar: 0.05 substitutions per nucleotide position. T: ex-type strains. (TIFF) [file pone.0112067.s002.tiff]
